# ScorpDb: A Novel Open-Access Database for Integrative Scorpion Toxinology

**DOI:** 10.3390/toxins16110497

**Published:** 2024-11-18

**Authors:** Masoumeh Baradaran, Fatemeh Salabi, Masoud Mahdavinia, Elaheh Mohammadi, Babak Vazirianzadeh, Ignazio Avella, Seyed Mahdi Kazemi, Tim Lüddecke

**Affiliations:** 1 Toxicology Research Center, Medical Basic Sciences Research Institute, Ahvaz Jundishapur University of Medical Sciences, Ahvaz 61357-15794, Iran; mahdavimasoud@yahoo.com (M.M.); e.mohammadi2626@gmail.com (E.M.); 2 Razi Vaccine and Serum Research Institute, Agricultural Research, Education and Extension Organization (AREEO), Ahvaz 31976-19751, Iran; fat_sa_2012@yahoo.com; 3 Department of Toxicology, School of Pharmacy, Ahvaz Jundishapur University of Medical Sciences, Ahvaz 61357-15794, Iran; 4 Social Determinant of Health Research Center, Ahvaz Jundishapur University of Medical Sciences, Ahvaz 61357-15794, Iran; 5 LOEWE Centre for Translational Biodiversity Genomics, Senckenberganlage 25, 60325 Frankfurt am Main, Germany; ignazio.avella@ime.fraunhofer.de; 6 Institute for Insect Biotechnology, Justus Liebig University of Giessen, Heinrich-Buff Ring 26-32, 35392 Giessen, Germany; 7 Animal Venomics Lab, Fraunhofer Institute for Molecular Biology and Applied Ecology, Ohlebergsweg 12, 35392 Giessen, Germany; 8 Zagros Herpetological Institute, P.O. No 12, Somayyeh 14 Avenue, Qom 37156-88415, Iran; kazemi_m1979@yahoo.com

**Keywords:** scorpion, scorpion venom peptides, venomics, taxonomy, database

## Abstract

Scorpion stings are a significant public health concern globally, particularly in tropical and subtropical regions. Scorpion venoms contain a diverse array of bioactive peptides, and different scorpion species around the world typically exhibit varying venom profiles, resulting in a wide range of envenomation symptoms. Despite their harmful effects, scorpion venom peptides hold immense potential for drug development due to their unique characteristics. Therefore, the establishment of a comprehensive database that catalogs scorpions along with their known venom peptides and proteins is imperative in furthering research efforts in this research area. We hereby present ScorpDb, a novel database that offers convenient access to data related to different scorpion species, the peptides and proteins found in their venoms, and the symptoms they can cause. To this end, the ScorpDb database has been primarily advanced to accommodate data on the Iranian scorpion fauna. From there, we propose future community efforts to include a larger diversity of scorpions and scorpion venom components. ScorpDb holds the promise to become a valuable resource for different professionals from a variety of research fields, like toxinologists, arachnologists, and pharmacologists. The database is available at https://www.scorpdb.com/.

## 1. Introduction

Scorpions (phylum Arthropoda, class Arachnida, order Scorpiones) are an ancient group of invertebrates that have diversified over approximately 400 million years [1,2]. So far, more than 2800 scorpion species have been identified and classified into 23 different families [3]. They are distributed almost globally, and are only absent from a few temperate countries, Antarctica, and some Pacific islands [4]. Scorpions occur in most biomes, but the hotspots of their diversity are in tropical and subtropical regions of the world. Their tremendous biodiversity, wide distribution, and evolutionary age testify to scorpions’ biological, evolutionary, and ecological success [4,5].

One of scorpions’ key adaptions that has contributed substantially to their evolutionary success is their venom [6]. Scorpions use their venom mostly to overpower their prey, primarily insects. When threatened, scorpions may also use venom to fend off potential predators and/or attackers, including humans. Therefore, scorpion sting and the resulting medical symptoms (i.e, scorpionism) emerged as a public health concern in several regions of the world, especially in South America, North Africa, and the Middle East [7,8]. The incidence of scorpion stings varies across different geographical regions and countries [9], with the highest frequency reported in Mexico, Brazil, and Iran [10,11]. Notably, scorpions have also been used in traditional medicine in some African and Asian countries [12,13], and recent studies on scorpion venom have shown that it contains valuable biological compounds with potential medicinal properties [12,14,15,16]. Scorpion venom is a heterogeneous mixture composed of different bioactive molecules, including antimicrobial peptides (AMPs), neurotoxins, cardiotoxins, nephrotoxins, and hemolytic toxins. Furthermore, it may contain a variety of enzymes, including phosphodiesterase, phospholipase, or hyaluronidase [17,18,19,20,21,22]. Currently, scorpion venoms are mainly employed to develop antivenoms for the treatment of scorpion stings, and in drug discovery applications [19,23].

Due to scorpion stings being a medical threat across the globe, a significant amount of research efforts are invested into scorpions and their venoms. However, with the recent advent of modern venomics technologies, especially next-generation sequencing (NGS) and proteomics, as well as biotechnology [24,25], supplemented by the development of open-source bioinformatics software, the biodiscovery from scorpion venom is accelerating substantially. Many studies have used high-throughput NGS technologies to obtain transcriptomic data from scorpion venom glands [22,26,27,28,29,30,31,32]. This has gradually increased the amount of sequence-based data of scorpions as non-traditional model organisms. Despite the increasing availability of transcriptomic and proteomic data on scorpion venom and the medical and translational importance of scorpions [33,34], a comprehensive database has not yet been created to collect and categorize this information.

Due to the methodological particularities of modern venomics and beyond, access to accurate and comprehensive data is critical to making informed decisions and guiding discoveries [35]. A number of publicly accessible databases, including UniProt (https://www.uniprot.org, accessed on 20 October 2023) and NCBI (https://www.ncbi.nlm.nih.gov, accessed on 20 October 2023), offer access to venom protein sequences from various taxa. These databases are commonly used as references for novel venom characterizations. However, they are not dedicated exclusively to venom proteins, and the information they contain is often uploaded in a disorganized manner. This makes it challenging to locate, filter, and extract relevant data from public databases, thereby increasing the difficulty and time required for researchers. In order to enhance data accessibility in the field of scorpions and scorpion venom compounds, we herein present a novel comprehensive database, ScorpDb. This web-based database platform has been created to simplify the search and acquisition of information about scorpion species, as well as the peptides and proteins identified in their venoms. ScorpDb has been meticulously designed to provide a user-friendly platform to easily navigate a wealth of information on scorpions and their venom components. This database management system offers many functionalities for storing and retrieving data. Its key features include support for comprehensive information, freedom of access, multiple device types, and efficient indexing. Additionally, ScorpDb possesses a user-friendly interface enabling convenient and intuitive interaction. The development of this continuously updated database, including the latest information about scorpions and their venoms, aims to foster expert collaboration by offering a seamless interface for sharing findings and contributing to an ever-growing knowledge repository.

## 2. Results

**Figure 1:**
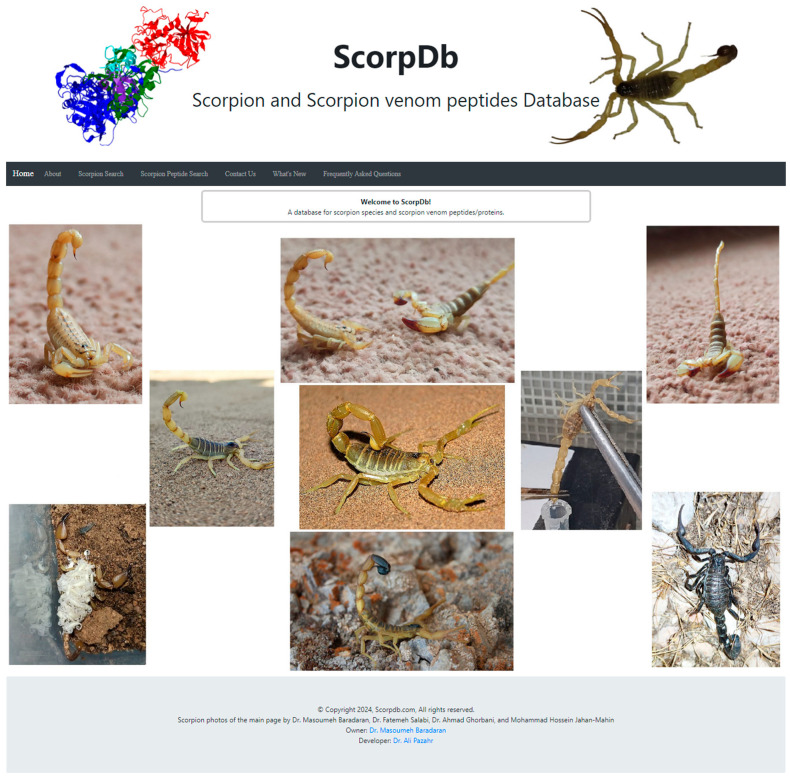
Main window of ScorpDb. The home page was considered as the initial page. It features seven tabs at the top of the page. Information about scorpion species and scorpion venom peptides is accessible by clicking on the two main tabs: “Scorpion Search” and “Scorpion Peptide Search”.

ScorpDb is a database for scorpion species and scorpion venom peptides/proteins. Figure 1 presents an overview of the main window of ScorpDb. As a starting point for the database, we opted to prioritize scorpions from our home country, Iran. This decision was motivated by the recent discovery that an optimized understanding of our local scorpion fauna is essential to inform the production of scorpion antivenoms [36,37]. Thus, we included the data currently available on venom, venom toxins, and natural history for Iranian scorpions. As an initial step towards depicting scorpion biodiversity, we supplemented the entries with high-quality pictures of important Iranian species, provided by our research group. For this purpose, different scorpion species were collected from all over Iran, transferred to our laboratories for identification based on their morphological properties [38], and photographed.

Likewise, we proceeded to include scorpions that do not live in Iran by implementing data and photographs from external sources. These were mainly retrieved from the websites iNaturalistGT (https://guatemala.inaturalist.org, accessed on 20 October 2023) and GBIF (https://www.gbif.org, accessed on 20 October 2023), and in some cases they were taken from articles which described the scorpion species of interest. Furthermore, we are currently seeking expert volunteers from different scorpion diversity hotspot countries that may take up curator positions and incorporate data on a broader diversity of scorpion species.

**Figure 2:**
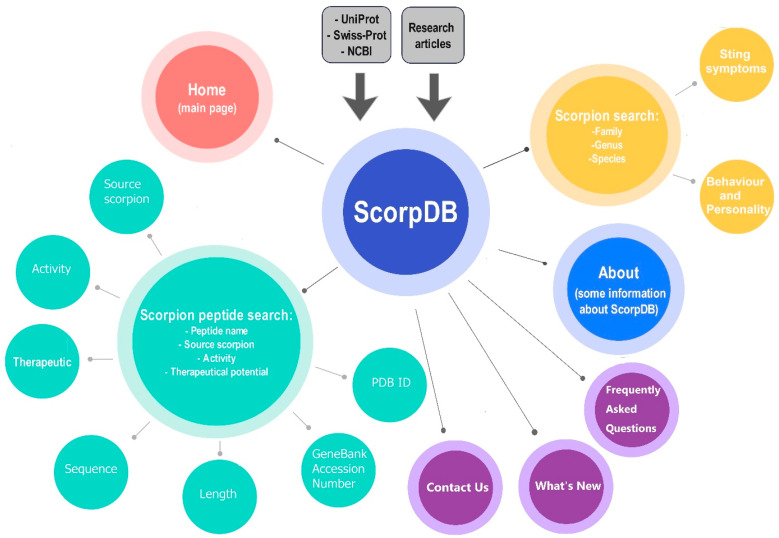
The overall architecture of ScorpDb. The main page of the site contains seven major tabs, which are connected to the central circle (ScorpDb) with dark gray lines in the image. The two main sections of the site, which contain information on the taxonomy of scorpions and scorpion peptides, are shown in green and yellow colors, respectively.

At the time of writing, the data collected in ScorpDb represent a starting point and will subsequently be refined and improved (particularly the photographic documentation and venom component overview) with future updates and releases of the database. The architecture of the ScorpDb database is given in Figure 2.

### 2.1. Web Interface of ScorpDb

**Figure 3:**
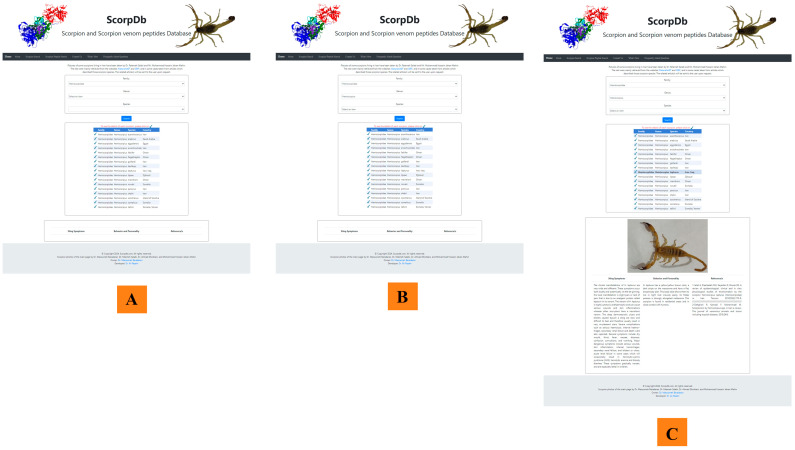
An overview of the user interface of the scorpion species search module of ScorpDb. (**A**) Searching output according to scorpion family. The entry *Hemiscorpiidae* is taken as an example. All genus and species of *Hemiscorpiidae* are shown in a table below the searching box. (**B**) Searches can be restricted by entering the name of the genus in the second box (Genus *Hemiscorpius* used as an example). (**C**) Alternatively, species can be selected from the third box (Species) or the list below (in the example, *Hemiscorpius lepturus*). By clicking on the tick mark next to the name of each scorpion species listed in the appearing table, additional information will be displayed in a separate box.

User interfaces play a crucial role in facilitating efficient and effective data management in online repositories [39]. In ScorpDb, the interface boasts a modern design incorporating clear visual elements and intuitive navigation, ensuring that users can quickly locate the tools they need. Upon logging in, pictures of scorpions can be seen. Users are greeted with a clean and intuitive menu that categorizes all data in seven navigation tabs at the top of the main page (Figure 1). Figure 3 provide a visual representation of the user interface of ScorpDb, offering an overview of its design and functionality. This figure enables users to gain insights into the range of functions available within the ScorpDb platform.

### 2.2. Major Features of ScorpDb

The user interface of ScorpDb is designed to enhance the user experience and facilitate efficient navigation. The major features of ScorpDb are outlined below.

*Searching facilities*: A user-friendly browsing interface has been created to retrieve information effortlessly. In the Scorpion search module, users can query the database by different taxonomic levels (Figure 3). Furthermore, the scorpion peptide search module allows users to search for information about any scorpion venom peptide using one or all of the following options: (i) peptide name; (ii) scorpion species from which it originates; and (iii) biological activity.

*Provide exhaustive information*: For each peptide, a web page containing a comprehensive dataset is available. This dataset includes the peptide’s name, the scorpion species producing it, the sequence and sequence length, as well as the type and activity of the peptide. Peptides with medicinal potential are presented along with details of their activity and effect on the type of disease. Furthermore, each scorpion species has a webpage on the site, providing detailed information about their location, prey spectrum, reproduction, epidemiology, and medical relevance, together with a picture of the species (Figure 3).

*Mobile website compatibility*: The ScorpDb web interface offers seamless functionality across multiple devices. This allows users to access the platform from desktops, laptops, or mobile devices without hindering usability. This ensures that users can stay connected and productive regardless of their location or preferred device.

*Include up-to-date information*: Scorpion taxonomy has undergone several changes and reorganizations over time, with the frequent description of new species and the renaming of others. As an example, *Mesobuthus eupeus*, locally known as “tosan” in Iran, has recently been subjected to taxonomic revision. In previous studies, *Mesobuthus eupeus* has been reported as one of the scorpions found in the southwest of Iran. However, a recently published study, in addition to reporting 14 new species of *Mesobuthus*, also points out that scorpions of the genus *Mesobuthus* in southwestern Iran belong to the species *Mesobuthus crucittii* [40], and not to *M. eupeus*. This highlights the importance of reporting data related to scorpions correctly, and dynamically curating the entries within linked repositories. In ScorpDb, we have integrated these data with the most recent available scorpion taxonomy, and will revise them following the most recent taxonomic changes to keep the databases updated.

## 3. Discussion

### 3.1. The Benefits of ScorpDB

Given that scorpion venom is a prolific resource of potential drug leads as well as a possible threat to human health worldwide, a large bulk of studies aimed to investigate its components. Recently, this has been accelerated by modern “-omics” approaches, alongside traditional pharmacology-guided studies. However, the lack of a comprehensive scorpion venom database represented a significant disadvantage for those investigations. In response to this need, we have developed and herein present ScorpDb, a novel database for scorpion species and venom components. This tool aims to provide a valuable foundation for studying and understanding scorpions and their toxic arsenals.

Due to the importance of scorpion stings and scorpion venom, some websites, such as the Scorpion Files (https://www.ntnu.no/ub/scorpion-files/, accessed on 20 October 2023), specifically collect and present information about different scorpion taxa. Additionally, other repositories like Arthropoda Cytogenetics (https://arthropodacytogenetics.bio.br/scorpiondatabase/, accessed on 20 October 2023), iNaturalistGT (https://guatemala.inaturalist.org, accessed on 20 October 2023), and GBIF (https://www.gbif.org, accessed on 20 October 2023) have implemented a number of scorpion-centric data lists. Despite being typically comprehensive and inclusive (i.e., allowing citizen scientists and researchers from less economically advantaged regions to easily submit data), these databases only provide taxonomic and natural history information about scorpions, and do not include information about peptides and other components present within their venoms.

Other repositories, such as SCORPION [41] and SCORPION2 [42], which collected information on scorpion toxins (although largely omitting taxonomic and natural history data), are no longer available. The Kalium database (https://kaliumdb.org/, accessed on 20 October 2023) provides information about scorpion potassium channel-modulating toxins (KTxs) [43], but does not include other, equally crucial toxins, including sodium channel toxins (NaTxs), chloride channel toxins (ClTxs), calcium channel toxins (CaTxs), and antimicrobial peptides (AMPs). Similarly, antimicrobial scorpion peptides are archived in various databases, such as AMPDB (https://bblserver.org.in/ampdb/, accessed on 20 October 2023) [44], AMPD3 (https://aps.unmc.edu/database/peptide, accessed on 20 October 2023) [45], APD (https://aps.unmc.edu/, accessed on 20 October 2023) [46], DRAMP (http://dramp.cpu-bioinfor.org/, accessed on 20 October 2023) [47], and DBASSP (http://dbaasp.org, accessed on 20 October 2023) [48], again omitting all other venom components. Through the ScorpDB database, in addition to all identified scorpion peptides (both toxic and non-toxic), we provide the first integrative platform also incorporating scorpion taxonomy, biology, and translational potential. This information not only provides users with details about scorpion venom peptides, but also offers insight into their medicinal potential.

### 3.2. Current Limitations and Strategies to Overcome Them

While the integrative database presented here conceptually represents a valuable resource to foster scorpion and scorpion venom research, it is important to consider a few limitations that need to be addressed.

Firstly, scorpions are a relatively diverse group of organisms that contain several thousand species and for many of them the taxonomic situation is ambiguous. Hence, it will be a major task to integrate a larger diversity of scorpions and scorpion peptides into the proposed database and to keep the selected array updated according to the most recent taxonomic assignments. Although we have already established categories for a large taxonomic fraction of scorpions, we were only able to enter data to the extent required for a few selected taxa. This primarily includes species from our country (Iran), together with entries from other species of the highest medical importance from other countries. We consider this as a good starting point and we will develop and grow it in future versions. This will require colleagues with special expertise in the missing scorpion lineages from other geographical areas to step up as curators and increase the breadth of taxa available. In this regard, it would be very attractive to also harness our implementation of photographic documentation to a much higher extent. At the time of writing, we have provided images of whole animals; however, for a precise taxonomic identification this is probably insufficient. Therefore, it would be favorable to also include detailed images of important morphological traits through which species can be identified.

The same applies to venom components, as for the vast majority of scorpions, except Buthidae, venom profiles are largely unknown. Therefore, in addition to growing the taxonomic diversity within ScorpDB, it will be pivotal to increase the molecular space within scorpion venoms alike and to funnel this information into our database. In that context, it is important to point out the need to submit raw data in venomic studies from which this type of information could be retrieved. Unfortunately, this is not always done to the extent necessary, as recently discussed in relation to the example of arachnids [49].

A final limitation of ScorpDB is that it is currently still somewhat restricted in terms of its level of popularity. However, we envision our database growing into a more complete and better supplied resource as, at the time of writing, it is solely based on the efforts of a small team and hence has plenty of room for growth. This includes the need to subsequently increase the array of available and cited literature and ensure a wider diversity of scorpion images, higher accuracy of clinical data, and updated distribution maps, amongst others. We also envision embedding cross references to other important databases, such as the world arachnid server or VenomZone, but have specifically considered including more advanced tools for toxin sequence analysis as well. Moreover, these updated versions will benefit tremendously from the efforts of future curators to be recruited, and will, finally, result in ScorpDB emerging as an important resource in the future.

## 4. Conclusions

Despite the tremendous scientific efforts invested into the study of scorpion venom and scorpionism, a comprehensive database for these topics has been lacking thus far. ScorpDB is a public and easy-to-access database which provides exhaustive information about scorpions’ taxonomy, biology, venom-derived peptides, and translational potential. Although we have so far prioritized scorpion species from Iran, a growing body of volunteering curators will subsequently increase the scope and extent of the repository beyond our home country. The creation of a specialized database filling this important gap has the potential to greatly benefit professionals from various research fields. By providing access to extensive, meticulously curated datasets, this database will enable researchers to access valuable insights into a plethora of relevant particularities needed to perform stringent toxinological studies on scorpions. This, in turn, may lead to advancements in the development of new drugs and therapeutic tools, and help to shed light on hitherto poorly understood aspects of scorpion biology. Thus, ScorpDb provides a valuable addition to the scorpion toxinological toolkit and beyond. However, to effectively achieve this important strategic goal, it will be necessary to engage in collaborative efforts. As it stands, the database is an early-stage project, and its curation mostly revolves around scorpions from Iran. Hence, a panel of scorpion and scorpion venom experts from key geographic regions needs to be recruited to establish and curate the relevant entries from around the world. Through such a community-wide concerted effort, it will be possible to establish ScorpDB as a global integrative resource for scorpion toxinology. If any scientist or expert is interested in joining the ScorpDB curatorial team, they can contact us through the following email: mb.baradaran@gmail.com. In addition, in the ‘Contact Us’ section of the website, the dedicated email related to site management can also be seen (Scorpdb.help@gmail.com).

## 5. Materials and Methods

### 5.1. Architecture and Implementation of ScorpDb

The ScorpDb website was developed using Microsoft Visual Studio.NET 2022. The programming language C# was used as the backend to implement the required specifications. The server-side technology, ASP.NET framework 4.8, was employed to generate dynamic web pages. For the frontend of the website, HTML, CSS, and JavaScript were used. Microsoft SQL Server 2019 was used as the database management system for storing and retrieving information. Due to the importance of portability and the need for the application to run correctly on different devices, bootstrap technology was implemented to create a responsive web design. All major browsers (e.g., Google Chrome, Microsoft Edge, and Mozilla Firefox) are supported. The server PC is located in Iran. Fanavari Serverpars Argham Gostar Company maintains the database. Toxicology Research Center of Ahvaz Jundishapur University of Medical Sciences is responsible for updating the database.

### 5.2. Setting Up the Database: Data Acquisition and Classification

The data required for preparing the database were divided into two main categories: (i) arachnological data and (ii) information on known scorpion venom components. The arachnological data (i) includes important taxonomic resources (e.g., family-level assignment of scorpions), as well as ecological, biogeographic, and clinical symptoms due to sting, and toxinological data. The taxonomic data for scorpions were retrieved from Scorpion Files [3]. Information on the various scorpion species was extracted from articles and books and is the result of the research undertaken by our research group at the Toxicology Research Center of Ahvaz Jundishapur University of Medical Sciences and the Razi Vaccine and Serum Research Institute. The information on known scorpion venom components (ii) was retrieved from public databases and in-house generated transcriptome data. Some data related to scorpion venom peptides and proteins were extracted from the Uniprot, Pfam, NCBI, and Swiss-Prot protein databases and combined with our group’s research. The search was conducted based on the names of the scorpion species, one by one, and all identified peptides included in the databases were entered into ScorpDb.

To search for scorpion peptides and proteins, in brief in the databases mentioned above, the proteins for each taxon were retrieved by using the species name of each scorpion as a search query. The retrieved protein sequences were further analyzed by InterproScan (https://www.ebi.ac.uk/interpro/search/sequence/, accessed on 20 October 2023) to identify potentially relevant candidates and to gather structural insights as well as to perform protein family assignments. The inclusion criteria for peptides and proteins were the existence of signal peptides and at least one domain related to scorpion venom proteins. All peptides and proteins without these criteria were excluded. Similarly, known cellular proteins and other proteins expressed by housekeeping genes were not further considered. Some of the peptides and proteins stem from our venom gland transcriptomes of scorpions from the Iranian Khuzestan province. The method of identifying scorpion peptides from the venom gland transcriptome has been described in our previous studies [22,50,51,52,53,54].

## Data Availability

Pictures of some scorpions living in Iran have been taken by Dr. Fatemeh Salabi, Mohammad Hossein Jahan-Mahin. The rest were mainly retrieved from the websites iNaturalistGT (https://guatemala.inaturalist.org, accessed on 20 October 2023) and GBIF (https://www.gbif.org, accessed on 20 October 2023), and in some cases taken from articles which described those scorpion species. The related article/s will be sent to the user upon request. Scorpion venom peptides and proteins were extracted from the Uniprot, Pfam, NCBI, and Swiss-Prot protein databases, and combined with our identified scorpion peptides and proteins. The information related to each peptide or protein is described on the ScorpDb website and will also be sent to the user upon request.
